# Impairment of cold injury-induced muscle regeneration in mice receiving a combination of bone fracture and alendronate treatment

**DOI:** 10.1371/journal.pone.0181457

**Published:** 2017-07-17

**Authors:** Shigeo Kawada, Atsushi Harada, Naohiro Hashimoto

**Affiliations:** 1 Department of Regenerative Medicine, Institute, National Center for Geriatrics and Gerontology, Morioka, Oobu, Aichi, Japan; 2 Department of Orthopedic Surgery, Hospital, National Center for Geriatrics and Gerontology, Morioka, Oobu, Aichi, Japan; University of Minnesota Medical Center, UNITED STATES

## Abstract

Alendronate, a nitrogen-containing bisphosphonate, is well established as a treatment for osteoporosis through regulation of osteoclast activity. Previously, the pharmacological effects of bisphosphonates on cells outside the bone environment have been considered irrelevant because bisphosphonates target bone. Here we show that administration of alendronate impairs muscle regeneration in mice after bone fracture. A series of injections of alendronate alone or bone fracture alone did not affect muscle regeneration induced by cold injury. In contrast, alendronate treatment plus bone fracture severely impaired the regeneration of muscle that closely contacts the bone fracture site after cold injury. After cold injury, M-cadherin-positive myogenic cells disappeared in the damaged muscle areas of mice receiving the combination of alendronate treatment and bone fracture. The present results suggest that the muscle regeneration capacity is impaired by bone fracture in mice receiving alendronate treatment. The present research on the pharmacological effects of alendronate on muscle regeneration will aid in understanding of the in vivo action of alendronate on skeletal muscles.

## Introduction

Bisphosphonates are non-metabolic synthetic analogues of pyrophosphate and well established as leading drugs for the treatment of osteoporosis and other diseases characterized by an increase in bone resorption [1, 2]. Bisphosphonates inhibit osseous resorption, depending on their two key properties: their high affinity to bone mineral and their inhibition of osteoclast activity. Their backbone structure, called P-C-P and containing two phosphate groups and a carbon atom, is required for both binding to bone mineral and antiresorptive activity [2]. The P-C-P moiety is involved in binding of bisphosphonates to hydroxyapatite, resulting in their selective uptake by the skeleton. During resorption, the acidic pH in the resorption space modifies one or both phosphate groups and dramatically reduces the affinity of bisphosphonates for bone mineral. Therefore, osteoclasts are exposed to a locally high concentration of bisphosphonates and preferentially internalize them. Osteoclasts are intended target cells of bisphosphonates because of the selective uptake of them, although cellular functions of osteocytes and other cells are also affected by bisphosphonates [3, 4].

Farnesyl pyrophosphate synthetase (FPPS), an enzyme of the mevalonate pathway, is a well-known target molecule of bisphosphonates [1, 2]. Bisphosphonates deplete isoprenoid lipids and geranylgeranyl diphosphate through inhibition of FPPS, and then prevent post-translational prenylation of small G proteins [1]. Inhibition of prenylation blocks the localization to plasma and subcellular membranes of small G proteins and attenuates their functional activity [5]. Small G proteins play essential roles in multiple cellular functions. Bisphosphonates are assumed to have similar impacts on proliferation, migration, cytoskeletal architecture, and signal transduction of various types of cell. It has been well-known that bisphosphonates act as antitumor reagents in tumor cells [6, 7]. In addition, untransformed cells, including macrophages, keratinocytes, and myoblasts undergo apoptosis when exposed to bisphosphonates [8–14]. The previous investigations strongly suggest that bisphosphonates exhibit cellular toxicity in various cell types other than osteoclasts.

Bisphosphonates belong to a specific drug class that is capable of highly specific interaction with osteoclasts because their ability to bind strongly to bone mineral allows them to be selectively taken up by bone. The bone-targeting property may minimize their effects on cells outside the bone environment in vivo despite their general cellular toxicity to various cell types. However, bisphosphonates may impair cells other than osteoclasts in vivo because they have been associated with a number of side effects [15]. Associations between adverse gastrointestinal events and oral administration of bisphosphonates and between acute phase reactions and their intravenous administration are well documented. Besides these side effects, chronic exposure to bisphosphonates may induce dysfunctions of non-skeletal tissues closely associated with bone. Skeletal muscle are tightly and closely connected to bone. Therefore, it is conceivable that skeletal muscle tissues are chronically exposed to bisphosphonates released from bone matrix during bone resorption in osteoporosis patients treated with bisphosphonates for many years. However, the previous studies provide controversial results concerning effects of bisphosphonates on skeletal muscles. Alendronate induces apoptosis of the rat myoblastic cell line L6 in vitro [10]. In contrast, alendronate does not affect skeletal muscle function of ovariectomized rats in vivo [16]. In addition, a clinical study shows that treatment with alendronate increases muscle mass in patients with osteoporosis [17].

We previously found that alendronate impairs functions of undifferentiated human myogenic cells but not terminally differentiated myotubes [12]. Therefore, we speculated that alendronate attenuates the regeneration capacity of muscle in vivo. In the present study, to determine whether alendronate reduces muscle regeneration potential, we induced muscle regeneration in mice treated with alendronate. We found that a combination of bone fracture and alendronate treatment prevented muscle regeneration after injury in mice. The present study will aid in understanding of the pharmacological effects of alendronate on skeletal muscle functions in vivo.

## Materials and methods

### Ethics statement

All animal experiments were approved by the National Center for Geriatrics and Gerontology Animal Ethics Committee (S1 File).

### Animals

Five-week-old male Swiss albino mice (Slc:ICR) were purchased from Shizuoka Laboratory Animal Center (Shizuoka, Japan) and used for all experiments. Normal and ovariectomized female mice were not analyzed in the present study to avoid plausible effects of estrogen on skeleton. All mice were housed under specific pathogen free conditions in groups of up to five mice per cage with 14 light and 10 h dark cycle and received a standard mouse feed and water. Three or four mice were examined in each experimental and control group at each time point as described in figure legends.

### Bone fracture

Open bone fracture was done as previously described [18]. Mice were anesthetized by inhalation of isoflurane. After scrubbing the right thigh, an 8-mm incision was made on the outside of the right thigh along the femur from the knee. The compartment between the quadriceps femoris and hamstring muscles was cut open using a surgical scalpel to visualize the femur. Then the mid portion of the femur was cut using a surgical scissors. After cutting the femur, the patella was dislocated to expose the femoral condyles. The intramedullary canal at the intercondylar notch was opened with a 0.5-mm diameter trephine. The tip of a 0.75-inch, 27-gauge needle was inserted directly into the medullary canal, up to the proximal end of the femur to fix the fracture, and the incision was sutured. No analgesics were administered. The same surgery was performed in the sham-operated group as a control, with the exception that the femur was not cut.

### Cold injury of vastus lateralis muscle

The lateral surface of the vastus lateralis muscle adjacent to the femur was exposed. The blunt end of a stainless steel spatula, measuring 3 mm x 4 mm and cooled in liquid nitrogen was applied to the exposed muscle for 10 seconds. Physical contact with a chilled microspatula caused local damage to the surface of the vastus lateralis muscle adjacent to femur bone.

### Bisphosphonate alendronate treatment

Alendronate (Sigma, St. Louis, MO) was dissolved at 100 mM in distilled water as a stock solution that was diluted with PBS immediately before administration. Mice were subcutaneously injected with alendronate (0.7 nmole per g body weight) every seven days. For control mice, the same volume of phosphate-buffered saline (PBS) was injected. The subcutaneous route of administration was the most convenient for the treatment of mice whereas the oral administration is the most popular route for alendronate in interventions.

### Combination of bone fracture and alendronate treatment

Mice were injected with alendronate or PBS three times at seven-day intervals and then were subjected to either cold injury of the muscle alone or both bone fracture and cold injury. For the “pulse and “chase” treatment of alendronate, mice in the control group were injected with PBS six times. In the “pulse” treatment group, mice were injected with alendronate three times before the operation, and then injected with PBS the last three times after the operation to avoid the proximate action of alendronate on regenerating skeletal muscle. Mice in the “chase” treatment group were injected with alendronate six times. Then the vastus lateralis muscles were removed from mice on day 42.

### Histopathological analysis

Mice were sacrificed by cervical dislocation. The area of the vastus lateralis muscle adjacent to intact bone or the bone fracture site was removed and pathologically analyzed. A small piece of muscle tissue was frozen in isopentane and cooled with liquid nitrogen as previously described [19–21]. Frozen muscle tissues were sectioned at a thickness of 7 μm with a cryostat (Leica Microsystems, Wetzlar, Germany), and then subjected to hematoxylin (Wako Chemical, Osaka, Japan) and eosin (Wako Chemical) staining. For immunofluorescence analysis, serial frozen sections were fixed with 4% paraformaldehyde for 5 min, incubated with rat monoclonal antibodies to CD31 (1:50; BD Pharmingen, Franklin Lakes, NJ) or F4/80 (1:100; AbD Serotec, Raleigh, NC), rabbit polyclonal antibodies to laminin α2 (1: 100 dilution; Sigma), or sheep antibodies to M-cadherin (1:200; R&D Systems, Minneapolis, MN) at 4°C overnight. They were then incubated with Cy3-labeled antibodies to rabbit IgG (Jackson ImmunoResearch Laboratories, Bar Harbor, ME) and Alexa Fluor 488-labeled antibodies to rat IgG or sheep IgG (Jackson ImmunoResearch Laboratories) for 1 h. The sections were mounted in SlowFade Gold antifade reagent with 4’,6-diamidino-2-phenylindole (DAPI) (Invitrogen, Carlsbad, CA). Samples were visualized using an upright microscope (model BX50; Olympus, Tokyo, Japan) and a CCD camera (DP70; Olympus).

For the quantification of muscle regeneration, number of peripherally nucleated myofibers (mature myofibers) and centrally nucleated myofibers (regenerating myofibers) were counted exclusively in the regenerating area of the muscle (1.0 x 0.2 mm). Notably, muscle areas far from the injured surface were not analyzed because they remained intact and did not undergo regeneration. The average and standard deviation were analyzed using one-way ANOVA.

## Results

### Bone fracture alone or combined bone fracture and alendronate does not cause muscle damage

**Fig 1:**
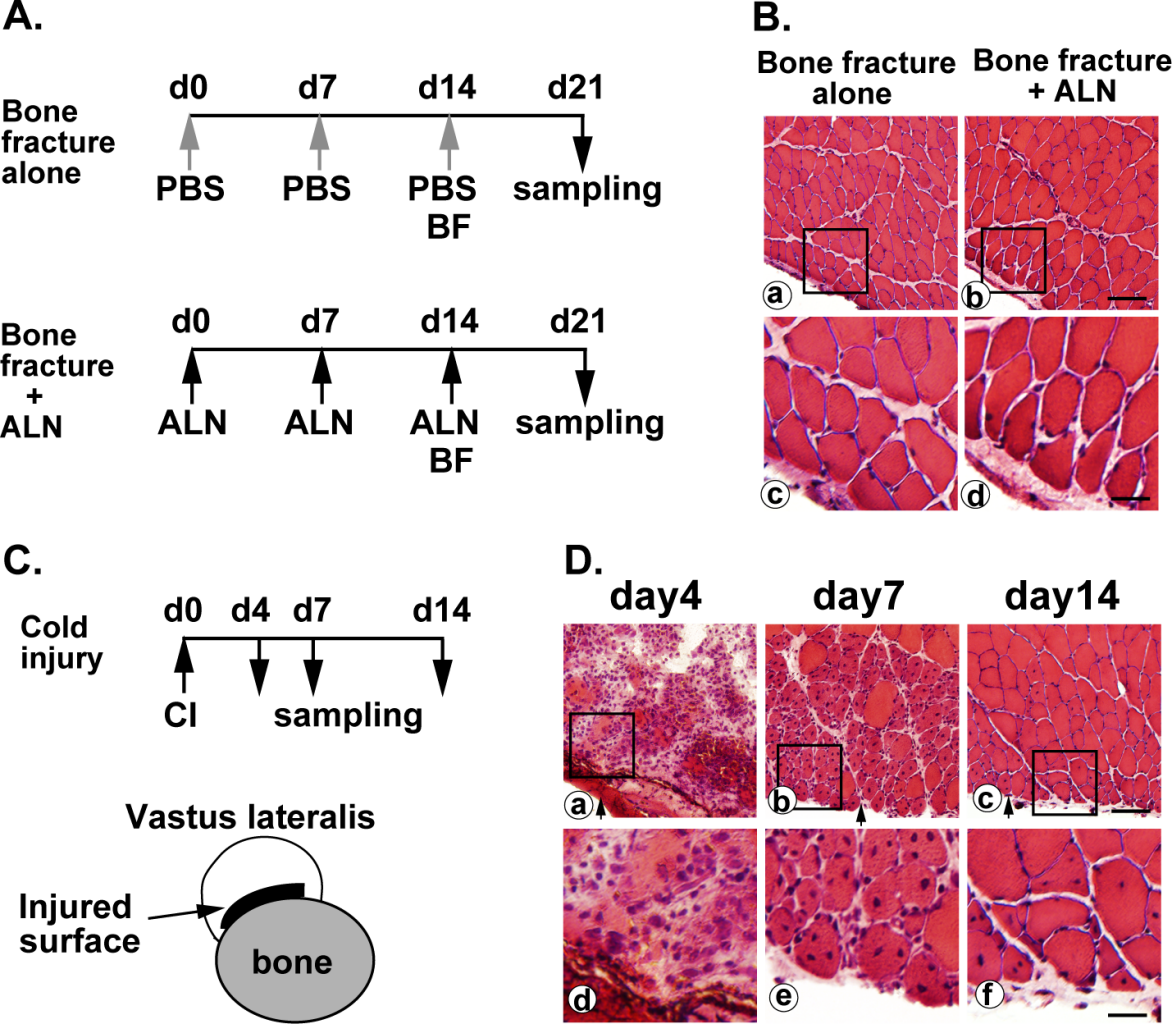
Effects of alendronate, bone fracture, and cold injury on muscle regeneration. (A) Experimental schedules. PBS (gray arrows) or alendronate (black arrows) was subcutaneously injected at seven-day intervals. The femur was broken on day 14 and removed on day 21. In the cold injury experiment, the vastus lateralis muscle was removed on day 4, 7, or 14 after a single cold injury. ALN, alendronate; BF, bone fracture. (B) Vastus lateralis muscles were sectioned and stained with hematoxylin and eosin. Squares in the top panels (a and b) indicate the areas magnified in the bottom panels (c and d). Scale bars, 60 μm in (a and b), 20 μm in (c and d). (C) Experimental schedule and schematic view of injured area of vastus lateralis. CI, cold injury. (D) Histopathology of muscle regeneration of vastus lateralis after cold injury. Arrows in (a-c) represent the surface injured by cold shock. Squares in the top panels (a-c) indicate the areas magnified in the bottom panels (d-f). Scale bars, 60 μm in (a-c), 20　μm in (d-f).

Alendronate binds strongly to the bone matrix and is released from bone during bone resorption [2]. In addition to resorption during bone turnover, femur bone was broken to induce the release of previously absorbed alendronate from bone matrix. First, we explored whether bone fracture alone or a combination of bone fracture plus alendronate-administration causes damage to muscles adjacent to a bone fracture site in mice (Fig 1A). On day 7 after the bone fracture, no sign of degeneration or regeneration was found in muscles of mice receiving the bone fracture alone (Fig 1Ba and c) or a combination of bone fracture plus alendronate treatment (Fig 1Bb and d). Consistent with our previous study using immortalized human myogenic cells in vitro [12], the results suggest that alendronate does not cause damage to myofibers even when mice receive a combination of bone fracture and alendronate treatment.

### Muscle regeneration after cold injury

To cause local damage to muscle areas adjacent to a bone surface, we chose a local cold injury method [22, 23]. First, we determined the process of regeneration of the vastus lateralis muscle after local cold injury. Only the damaged areas of muscles damaged by a single cold injury (within 0.2 mm from the injured surface) were pathologically analyzed on days 4, 7, and 14 (Fig 1C). Areas of muscles more than >0.2 mm from the cold injured surface remained intact throughout experiments. Myofibers had a necrotic morphology, prominent inflammatory reactions were induced, and many immune cells had infiltrated into the damaged areas of the vastus lateralis muscle on day 4 after the injury (Fig 1Da and d). On day 7, many regenerated myofibers having nuclei in the center of the cytoplasm, called centrally nucleated myofibers, were found in the damaged areas (Fig 1Db and e). The number of centrally nucleated myofibers decreased and the size of the myofibers became larger until 14 days after the injury (Fig 1Dc and f). The process of muscle regeneration after the local cold injury was similar to that of muscle regeneration induced by other methods [24].

### Combination of bone fracture and alendronate treatment prevents muscle regeneration

**Fig 2:**
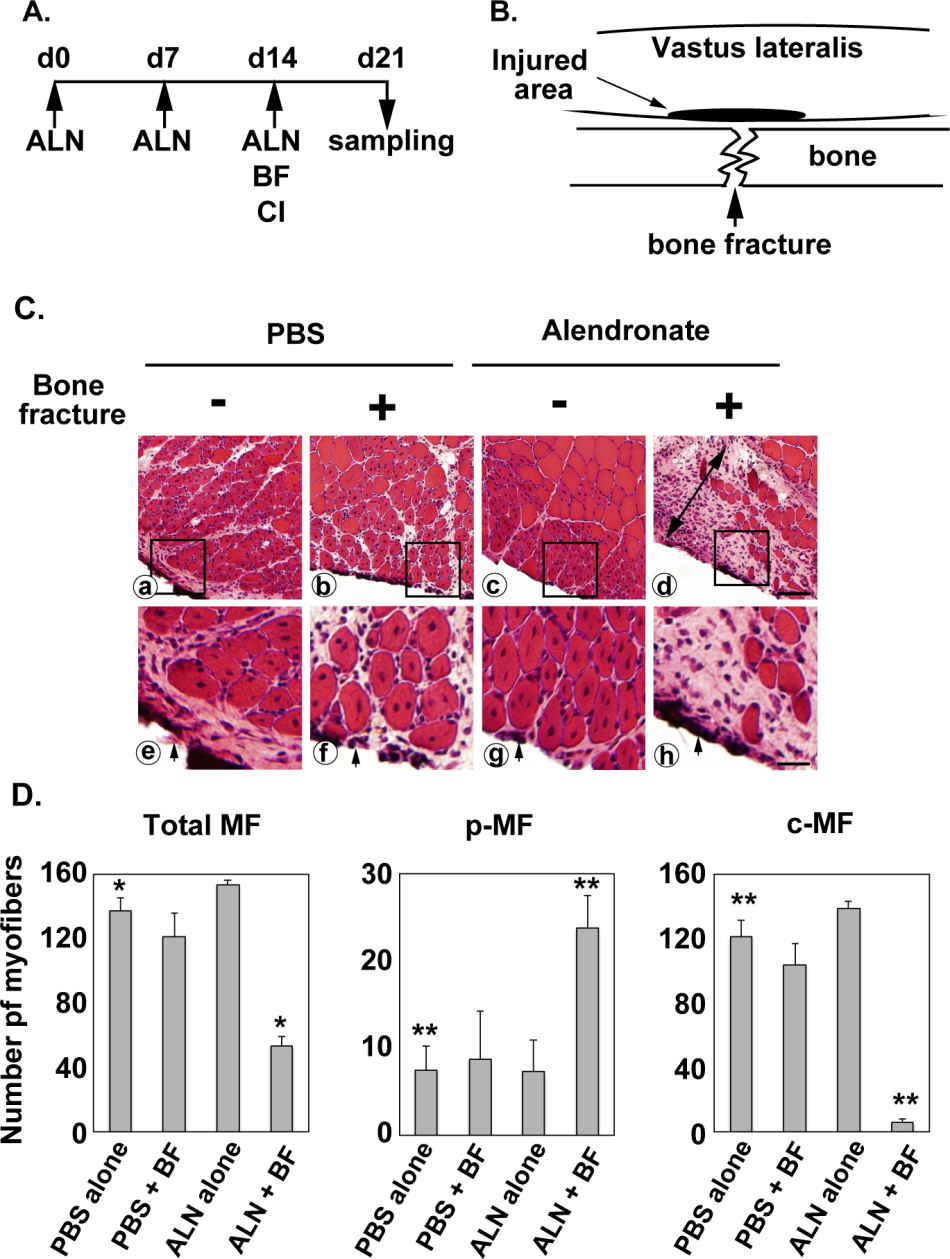
Combination of bone fracture and alendronate treatment prevents muscle regeneration. (A) Experimental schedules. Alendronate or PBS was subcutaneously injected at seven-day intervals. The femur was broken and the vastus lateralis muscle was injured by cold shock on day 14, and then the vastus lateralis muscle was removed on day 21. ALN, alendronate; BF, bone fracture; CI, cold injury. (B) Schematic view of the fracture site of femur and injured area of the vastus lateralis. (C) Histopathology of muscle regeneration of vastus lateralis after combination of bone fracture and muscle cold injury. A double-headed arrow in (d) represents the scar. Arrows in (e-h) represent the surface injured by cold. Squares in the top panels (a-d) indicate the areas magnified in the bottom panels (d-f). Scale bars, 60 μm in (a-c), 20 μm in (e-h). Numbers of peripherally nucleated myofibers (p-MF) and centrally nucleated myofibers (c-MF) were counted in the regenerating area (1.0 x 0.2 mm) of three independent samples. “Total” means the sum of the two types of myofibers. The average and standard deviation were estimated. Statistical significance (P-value) of difference between muscles of PBS-injected mice without bone fracture and each experimental group was analyzed using ANOVA. *, p<0.05; **, p<0.01.

Our previous study suggested that the regeneration capacity of muscle may be attenuated in osteoporosis patients by long-term alendronate treatment [12]. To uncover the effects of alendronate on muscle regeneration in vivo, we determined the effects of bone fracture alone, alendronate treatment alone, and a combination of bone fracture and alendronate treatment on muscle regeneration induced by cold injury (Fig 2A and 2B). Many centrally nucleated myofibers were reconstituted in muscles of PBS-injected mice without bone fracture on day 21 (7 days after cold injury) (control group) (Fig 2Ca and e). Bone fracture alone did not affect muscle regeneration induced by cold injury (Fig 2Cb and f). Alendronate treatment alone did not show any effect on muscle regeneration either (Fig 2Cc and g). In sharp contrast, the combination of bone fracture and alendronate treatment prevented muscle regeneration (Fig 2Cd and h). Intact myofibers were absent in the scar tissue of damaged areas (Fig 2Cd). Centrally nucleated myofibers were rarely found in the scar (Fig 2Ch). Quantitative analyses showed that the total number of myofibers and number of centrally nucleated myofibers significantly declined exclusively in muscles of mice receiving a combination of bone fracture and alendronate treatment (Fig 2D). The results suggest that alendronate attenuates the regeneration capacity of skeletal muscle exclusively upon bone fracture.

Small-sized peripherally nucleated myofibers remained in regenerating areas of the control and three experimental groups (Fig 2C). They showed the cytoplasmic basophilia that characterizes degenerating myofibers that appeared during the course of muscle repair. These small-sized basophilic myofibers with peripherally located nuclei were rarely found in uninjured muscle (e.g. Fig 1B). The number of these peripherally nucleated myofibers that remained in mice receiving the combination of bone fracture and alendronate treatment was significantly higher than those in the other groups. (Fig 2D, p-MF). The results suggest a possibility that a combination of bone fracture and alendronate treatment also attenuates phagocytosis of damaged myofibers by macrophages.

### Combination of bone fracture and alendronate treatment causes irreversible loss of muscle regeneration potential

**Fig 3:**
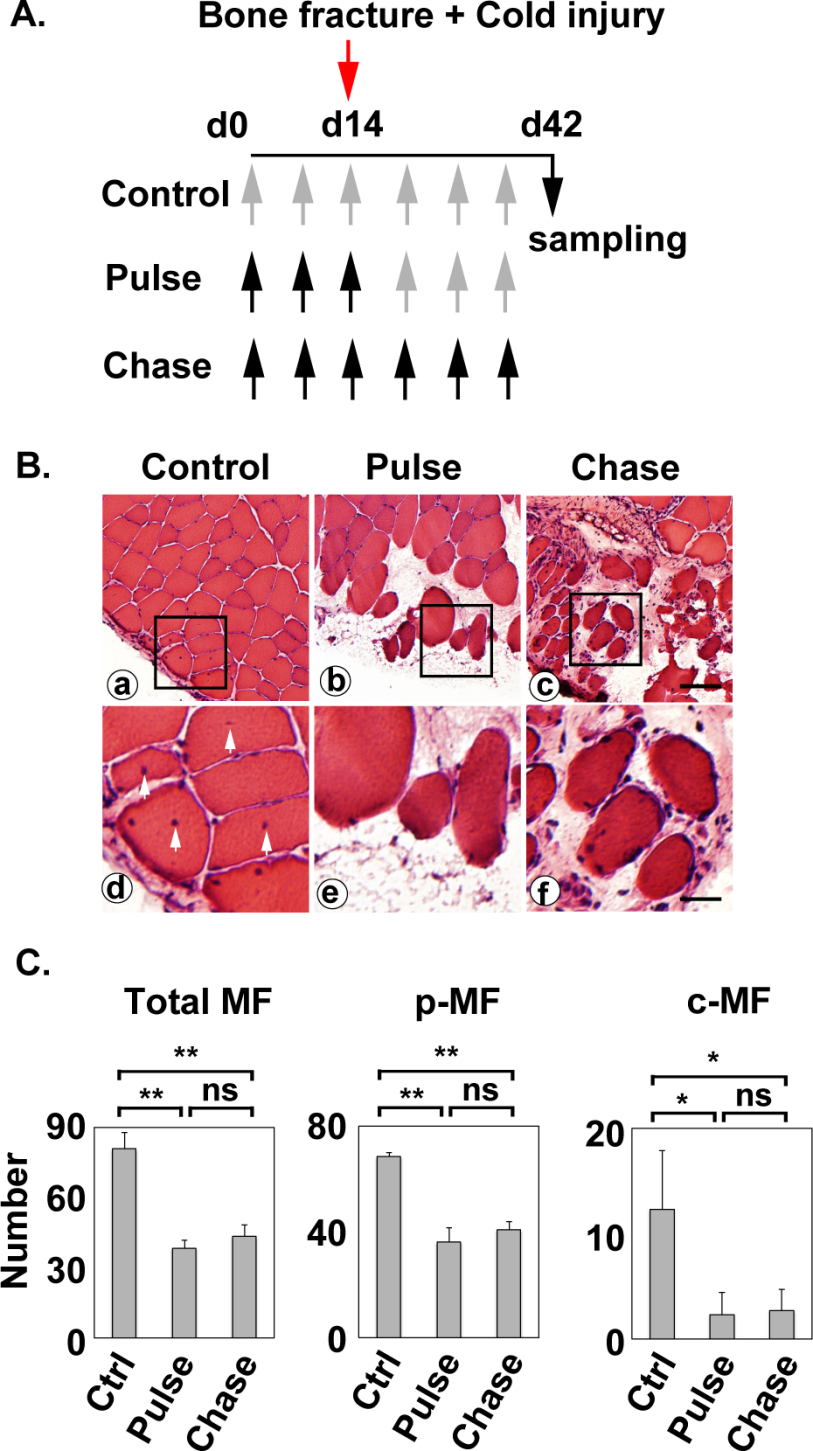
Combination of bone fracture and alendronate treatment causes irreversible loss of muscle regeneration potential. (A) Experimental schedules. PBS (gray arrows) or alendronate (black arrows) was subcutaneously injected at seven-day intervals. The femur was broken, and the vastus lateralis muscle was injured by cold shock on day 14, and then the vastus lateralis muscle was removed on day 42. (B) Histopathology of muscle regeneration of vastus lateralis. Control group (a and d), “Pulse” (b and e) and “Chase” (c and f) experimental groups are shown at low (a-c) and high (d-f) magnifications. Squares in the top panels (a-c) indicate the areas magnified in the bottom panels (d-f). Arrowheads represent the centrally located nuclei. Scale bars, 60 μm in (a-c), 20 μm in (d-f). (C) Numbers of peripherally nucleated myofibers (p-MF) and centrally nucleated myofibers (c-MF) were counted in the regenerating area (1.0 x 0.2 mm) of three independent samples. “Total” means the sum of both types of myofibers. The average and standard deviation were estimated. Statistical significance (P-value) of difference between muscles of control group and “Pulse” or “Chase” group, or muscles of “Pulse” group and “Chase” group was analyzed using one-way ANOVA. ns, p>0.05; *, p<0.05; **, p<0.01.

The next series of experiments were done to determine whether the muscle regeneration potential is recaptured after the cessation of alendronate treatment as described in “Materials and Methods” (Fig 3A). Mice in the control group were injected with PBS six times. In the “pulse” treatment group, mice were injected with alendronate three times before the operation, and then injected with PBS the last three times after the operation to avoid the proximate action of alendronate on regenerating skeletal muscle. Mice in the “chase” treatment group were injected with alendronate six times. Then the vastus lateralis muscles were removed from mice on day 42. Centrally nucleated myofibers were found in the control but their number significantly declined in both "pulse" and "chase" experimental groups (Fig 3B and 3C). Myofibers were reconstituted and large peripherally nucleated myofibers appeared in the damaged areas of muscle adjacent to the bone fracture site in the control group (Fig 3Ba and d). These peripherally nucleated myofibers did not show the cytoplasmic basophilia. In the “pulse” treatment group, a scar with extensive spaces between small-sized peripherally nucleated myofibers in the injured area of the muscle remained, although mice had not been treated with alendronate for 28 days after the cold injury (Fig 3Bb and e). The injured area of muscle remained damaged in the “pulse” treatment group (Fig 3Bb and e), as shown in the “chase” treatment group (Fig 3Bc and f).

Both the total number of myofibers and number of peripherally nucleated myofibers significantly declined in muscles of the “pulse” and “chase” treatment groups (Fig 3C). However, the total number of myofibers and number of peripherally nucleated myofibers were not significantly different between the “pulse” and “chase” treatment groups (Fig 3C). Peripherally nucleated myofibers found in in muscles of the “pulse” and “chase” treatment groups were degenerating myofibers that were small-sized and basophilic, whereas large-sized peripherally nucleated myofibers in the control group were completely regenerated.

The results indicate that treatment with alendronate prior to bone fracture and cold injury is sufficient to prevent muscle regeneration. The cessation of alendronate treatment did not promote the recovery of muscle regeneration potential. Therefore, pre-treatment with alendronate is sufficient to cause the irreversible loss of muscle regeneration potential in mice receiving bone fracture.

### Combination of bone fracture and alendronate treatment causes irreversible loss of myogenic cells at damaged areas of muscle

**Fig 4:**
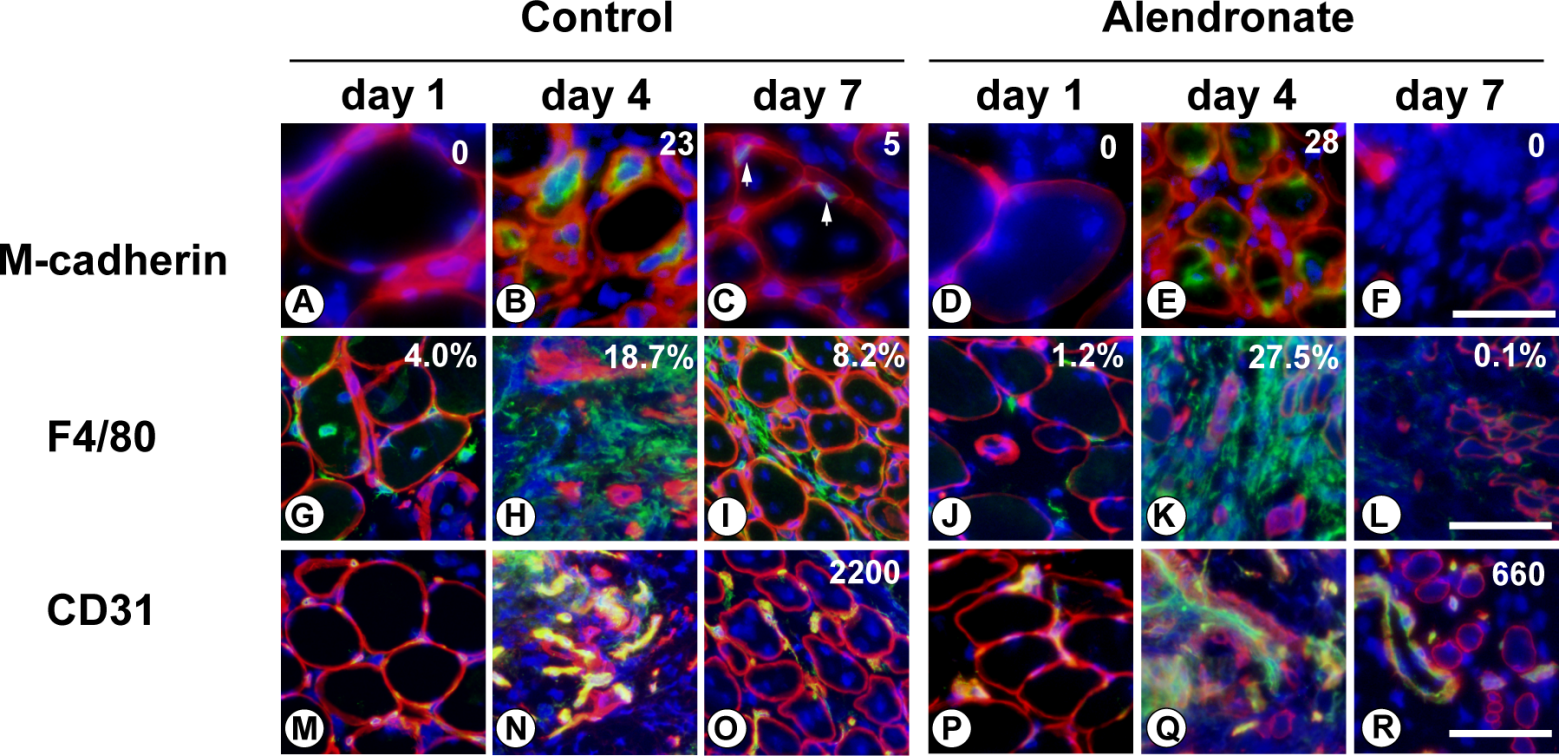
Combination of bone fracture and alendronate treatment causes loss of myogenic cells. PBS (Control group) or alendronate (Alendronate group) was subcutaneously injected three times at seven-day intervals. The femur was broken and vastus lateralis muscle injured by cold shock on day 14. Then the vastus lateralis muscle was removed on day 1, 4, or 7 after the bone fracture and cold injury. Sections were subjected to immunostaining with antibodies to M-cadherin (green in A-F), F4/80 (green in G-L), CD31 (green in M-R), and laminin α2 (red in A-R). Nuclei were stained with DAPI (blue in A-R). Arrows represent M-cadherin-positive muscle satellite cells in (C). Scale bars, 40 μm in (A-F), 20 μm in (G-R). Images represent a part of each original picture. Numbers represent numbers of M-cadherin-positive muscle satellite cells/myogenic progenitor cells in each original picture (0.17 mm^2^) in (A-F). Percentages of F4/80-positive cell-occupied area of each original picture (0.68 mm^2^) are shown in (G-L). Numbers of CD31-positive capillaries per mm^2^ of each original picture are shown in (O and R).

The postnatal growth, repair, and maintenance of skeletal muscle rely on muscle satellite cells, which play an essential role as skeletal muscle stem cells [21, 24]. To explore the role of muscle satellite cells in the impairment of muscle regeneration caused by alendronate, we preformed immunofluorescence analyses. M-cadherin is a muscle-specific classic cadherin that is expressed exclusively in the muscle satellite cell lineage [25]. In vastus lateralis muscles from mice receiving the bone fracture but not alendronate treatment, M-cadherin-positive muscle satellite cells were not found in the damaged region on day 1 after cold injury (Fig 4A). On day 4, M-cadherin-positive myogenic progenitor cells appeared around necrotic myofibers (Fig 4B). The myogenic cells were supposed to migrate from intact areas of muscles [24]. Then regenerating myofibers having centrally located nuclei were formed, and M-cadherin-positive muscle satellite cells were observed adjacent to the plasma membrane of myofibers beneath the basement membrane on day 7 after cold injury (Fig 4C). Muscle satellite cells disappeared on day 1, then myogenic progenitor cells migrated from intact areas into the damaged region on day 4 in muscles of mice receiving the combination of bone fracture and alendronate treatment (Fig 4D and 4E). In contrast to controls, myofibers were not reconstituted and M-cadherin-positive cells disappeared again in the damaged areas even after seven days (Fig 4F). The results suggest that myogenic progenitor cells accumulated in damaged areas but did not differentiate into myofibers in the injured area of muscles in mice receiving the combination of bone fracture and alendronate treatment. Then the myogenic progenitor cells may be damaged and lost.

Recently, there has been progress in understanding of roles of macrophages and endothelial cells during muscle regeneration [26, 27]. Therefore, we investigated whether macrophages and endothelial cells are involved in the defects in muscle regeneration caused by the combination of bone fracture and alendronate treatment. Macrophages were identified by F4/80 antibody. In control muscles of mice receiving the bone fracture but not alendronate treatment, macrophages were associated with necrotic myofibers and phagocytosed damaged myofibers on day 1 after cold injury (Fig 4G). The number of macrophages increased until four days after the muscle injury (Fig 4H). Then macrophages were still associated with juvenile myofibers in the regenerating areas, although their number decreased (Fig 4I). When vastus lateralis muscles of mice receiving the combination of bone fracture and alendronate treatment were injured, infiltration of macrophages was delayed on day 1 after the injury (Fig 4J). Thereafter the number of infiltrated macrophages increased until four days after injury (Fig 4K). However, macrophages had completely disappeared in the damaged areas on day 7 after the injury (Fig 4L). None of the hallmarks of regeneration was detected, and necrotic myofibers persisted until seven days after the injury. The results suggest that infiltration and/or survival of macrophages is inhibited in an alendronate treatment-dependent manner.

CD31-positive endothelial cells were associated with basement membrane that was labeled with anti-laminin α2 antibody and stimulated to proliferate until day 4 after muscle injury in the regenerating muscles of mice receiving the bone fracture without alendronate treatment (Fig 4M and 4N). Then capillary density was recovered until seven days after the injury (Fig 4O). In the muscles from mice receiving a combination of bone fracture and alendronate treatment, endothelial cells were also activated during the four days after the injury (Fig 4P and 4Q). However, the distribution of CD31-positive capillaries was not recovered at the damaged areas of muscles even on day 7 after the injury (Fig 4R). Neoangiogenesis was impaired in the regenerating muscles of mice treated with alendronate.

The present study suggests that a combination of alendronate-treatment and bone fracture prevents regeneration of adjacent muscle through impairment of multiple types of cells including myogenic progenitor cells, macrophages, and endothelial cells.

## Discussion

Locomotive ability largely depends on the function of bone and skeletal muscle. Thus, the dysfunction of bone and/or skeletal muscle gives rise to a loss of ambulation capacity, which is a major problem for the quality of life. Osteoporosis is currently estimated to be a major public health risk because it leads to bone fragility and fractures. Bisphosphonates have long been established as effective antiresorptive reagents for prevention and treatment of osteoporosis. Bisphosphonates target calcified tissues and are retained in bone for long periods. Then they are released from bone matrix and internalized by bone-resorbing osteoclasts. Forty-four percent of bisphosphonate alendronate is not taken up and retained in the skeleton but excreted in the urine 24 hours after administration [2]. Therefore, side effects of bisphosphonates that have been well-documented in oral and intravenous administration are thought to involve the proximate action of the circulating bisphosphonates on esophagus epithelial cells, γδT cells, and others [15]. Much bisphosphonate is rapidly buried within the mineral phase and retained for long periods within the skeleton, where it is pharmacologically inactive until the release from bone upon remodeling and resorption. Therefore, the pharmacological effects of bisphosphonates on cells outside the bone environment has remained to be examined in osteoporosis patients treated with bisphosphonates for many years. The chronic effects of bisphosphonates on neighboring tissues of bones should be determined during long-term bisphosphonate treatment. Skeletal muscles are possibly exposed to bisphosphonates that are released from bone matrix during bone remodeling and resorption in osteoporosis patients treated with bisphosphonates. However, the present study shows that circulating and released alendronate alone did not affect skeletal muscle closely connected to bone.

Our previous study showed that alendronate impairs proliferation and differentiation of undifferentiated human myogenic cells but does not affect the phenotype of terminally differentiated human myotubes [12]. Thus, we speculate that muscle regeneration capacity could be attenuated in vivo when treated with alendronate because undifferentiated myogenic cells, which play essential roles in muscle regeneration, are supposed to be susceptible to alendronate.

The present study suggests that muscle regeneration capacity is severely impaired in mice receiving a combination of bone fracture and alendronate treatment. The mechanisms by which alendronate inhibits muscle regeneration remains unexplained because there are multiple factors that affect muscle regeneration of mice receiving a combination of bone fracture and alendronate treatment.

First, we postulated that a proximate action of alendronate on myogenic cells causes inhibition of muscle regeneration. Concentration of alendronate may be higher in circulation when administrated. However, alendronate alone did not attenuate muscle regeneration potential. Therefore, myogenic stem cells called muscle satellite cells that are relevant to muscle regeneration may be resistant to alendronate as well as terminally differentiated myofibers [12]. Muscle satellite cells are activated upon muscle injury and turn to proliferating myogenic progenitor cells (myoblasts) [24]. In sharp contrast to muscle satellite cells and myofibers, our previous study suggests that proliferating myogenic progenitor cells are susceptible to alendronate in vitro [12]. Despite that, myogenic progenitor cells migrated from intact regions into damaged regions of muscle after cold injury even in mice receiving a combination of alendronate treatment and bone fracture. Therefore, circulating alendronate did not induce dysfunction of myogenic progenitor cells. In addition, if previously absorbed alendronate is released from bone matrix during remodeling after bone fracture, its concentration will be very low since function of osteoclasts may be severely attenuated during pretreatment with alendronate. Collectively, a proximate action of alendronate on myogenic cells is unlikely to be involved in the inhibition of muscle regeneration in mice receiving a combination of bone fracture and alendronate treatment.

Present results suggest that the possible indirect action of alendronate on skeletal muscle is involved in prevention of muscle regeneration. Angiogenesis by endothelial cells/endothelial progenitor cells and phagocytosis by macrophages were attenuated in regenerating muscle of mice receiving a combination of bone fracture and alendronate treatment. Pretreatment with alendronate may directly affect the properties of endothelial cells, circulating endothelial progenitor cells, and macrophages because these types of cell are directly exposed to circulating alendronate. Then, the putative damages to them may reduce their pool sizes and/or attenuate their cellular functions: blood capillary regeneration potential of endothelial cells/endothelial progenitor cells, and phagocytotic activity of macrophages. Impairment of functions of these cells may cause defects in muscle regeneration because regeneration of capillaries and phagocytosis of necrotic myofibers are essential to muscle regeneration.

Effects of bone fracture on muscle regeneration capacity remains undissolved. The putatively latent damage in endothelial cells/endothelial progenitor cells and macrophages may be not serious enough to reduce the muscle regeneration potential because pretreatment with alendronate alone did not prevent muscle regeneration caused by cold injury. Bone fracture probably triggers recruitment of endothelial progenitor cells and macrophages to a broken bone because bone repair is accompanied by repair of blood capillaries and an inflammation in osseous tissue. Cold injury on muscles also disrupts blood capillaries and triggers invasion of macrophages. We assume that recruitment of endothelial progenitor cells and macrophages to a broken bone may decrease the recruitment of these cells to the injured site of adjacent skeletal muscle because of the reduction in their own pool size by pretreatment with alendronate. If that is indeed the case, regeneration of the adjacent muscle may be seriously inhibited.

In addition, soluble factors from bone possibly affect muscle regeneration since bisphosphonates induce osteoblasts and bone marrow mesenchymal stem cells to produce paracrine factors [28, 29]. How and what paracrine factors from osteoblasts reach myogenic cells is still an open question.

The mechanism of prevention of muscle regeneration by alendronate treatment after bone fracture still remains puzzling. However, the present results suggest a possible impairment of muscle regeneration potential in patients receiving a combination of alendronate treatment and bone fracture.

In contrast to the present result, clinical reports have not shown defects of muscle regeneration in osteoporosis patients treated with alendronate. In addition, a recent clinical study indicated that alendronate increases the muscle mass of osteoporosis patients [17]. We should note that the direct impact of alendronate on myogenic cells highly depends on its dosage. Although high concentrations of alendronate (>100 μg/ml) prevent proliferation and differentiation of human myogenic progenitor cells, alendronate has no effect at lower concentrations. Myogenic progenitor cells are rarely exposed to high concentration of alendronate because circulating alendronate is taken up soon by bone. The present study suggest that bone fracture plays a role in prevention of muscle regeneration of adjacent muscle. Even when the traumatic injury induces both bone fracture and muscle regeneration in patients, the regenerating area of muscle may be not very close to the bone fracture site in most cases. Therefore, it is conceivable that the bone fracture is rarely accompanied with the loss of muscle regeneration potential in osteoporosis patients treated with alendronate. In addition, the loss of myofibers does not always decrease the muscle mass because the tissue space lacking myofibers is filled with non-myogenic/fibroblastic cells.

Our studies show that terminally differentiated myotubes/myofibers retain their function even when exposed to alendronate. Therefore, dysfunction of skeletal muscle need not be considered a limiting factor of alendronate treatment. In addition, we cannot exclude a possibility that the effects of alendronate on myogenic cells and muscle regeneration may be different from those of other species of bisphosphonates because each bisphosphonate has a unique profile and clinical effect [2].

Osteoporosis patients are usually treated with alendronate for many years. Hence, the chronic effects of alendronate on non-skeletal tissues and indirect effects on regeneration potential of skeletal muscle remain to be elucidated.

## Conclusions

Dysfunction of skeletal muscle is not a limiting factor of alendronate treatment because the function of uninjured skeletal muscles was not impaired by alendronate treatment alone. In contrast, muscle regeneration was prevented in mice receiving a combination of alendronate treatment and bone fracture. The results suggest a possible impairment of muscle regeneration potential in patients receiving a combination of alendronate treatment and bone fracture. The present study will contribute to understanding the possible effects of bisphosphonate on myogenic cells outside the bone.

## Supporting information

S1 File ARRIVE guidelines checklist. (DOCX)
