# Supplementary material for: Impairment of cold injury-induced muscle regeneration in mice receiving a combination of bone fracture and alendronate treatment
Source: PLoS One. 2017 Jul 17;12(7):e0181457. doi: 10.1371/journal.pone.0181457 (PMC5513540; doi:10.1371/journal.pone.0181457)

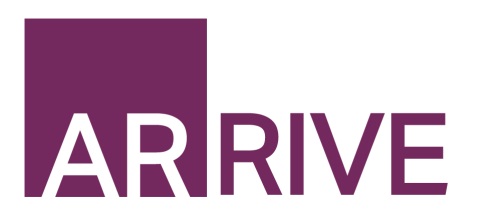


The ARRIVE Guidelines Checklist

Animal Research: Reporting In Vivo Experiments

Carol Kilkenny^1^, William J Browne^2^, Innes C Cuthill^3^, Michael Emerson^4^ and Douglas G Altman^5^

*^1^The National Centre for the Replacement, Refinement and Reduction of Animals in Research, London, UK, ^2^School of Veterinary Science, University of Bristol, Bristol, UK, ^3^School of Biological Sciences, University of Bristol, Bristol, UK, ^4^National Heart and Lung Institute, Imperial College London, UK, ^5^Centre for Statistics in Medicine, University of Oxford, Oxford, UK.*

|  | | ITEM | RECOMMENDATION | Section/ Paragraph |
| --- | --- | --- | --- | --- |
| 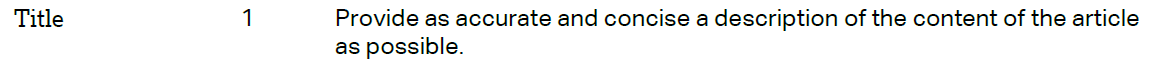 | | | Title |  |
| 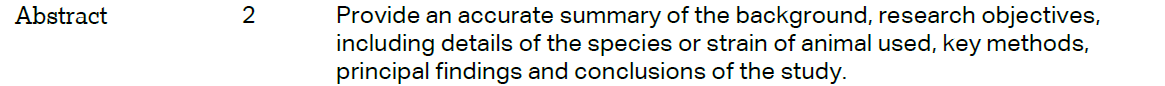 | | | Abstract |  |
| INTRODUCTION | | |  |  |
| 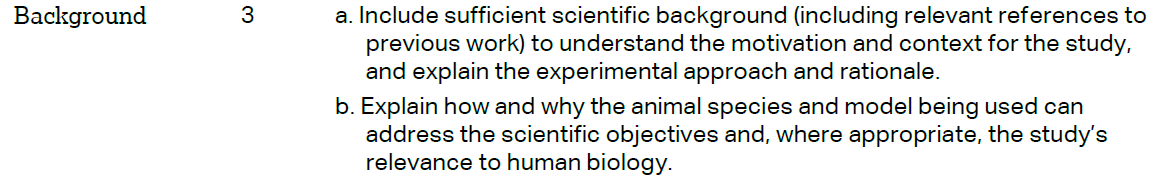 | | | Paragraph 1, 2  Paragraph 3 |  |
| 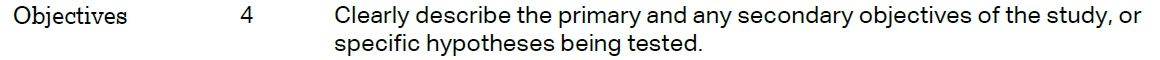 | | | Paragraph 4 |  |
| METHODS | | |  |  |
| 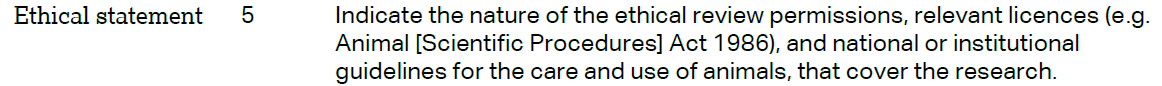 | | | Paragraph 1 |  |
| 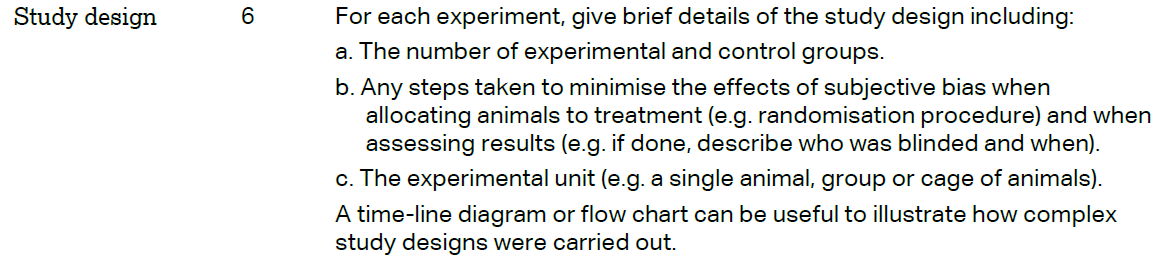 | | | Paragraph1, 2, 3, 4, 5  Figures 1-4 |  |
| 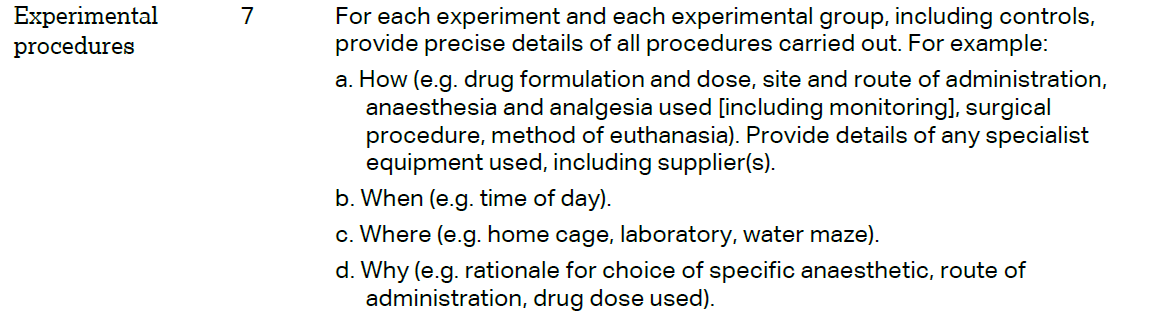 | | | Paragraph 3, 4, 5, 6, 7 |  |
| 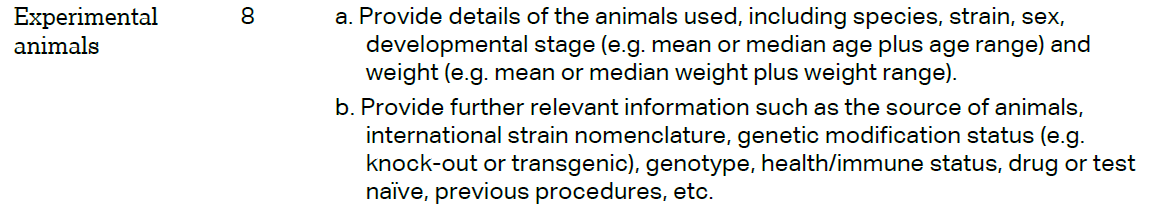 | | | Paragraph 1,2 |  |

The ARRIVE guidelines. Originally published in *PLoS Biology*, June 2010^1^

| 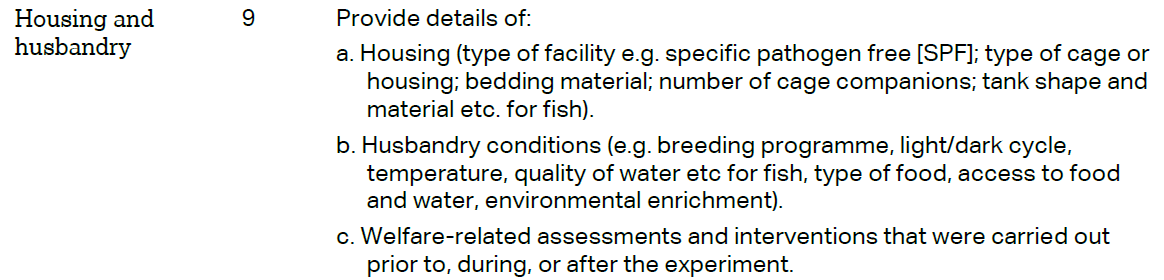 | Paragraph 2 |  |
| --- | --- | --- |
| 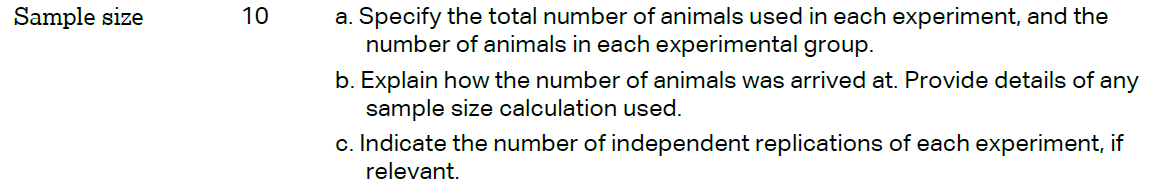 | Paragraph 2 |  |
| 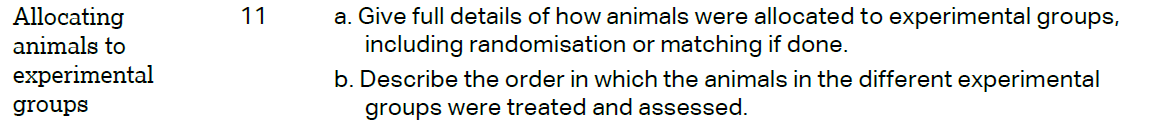 | Paragraph 2 |  |
| 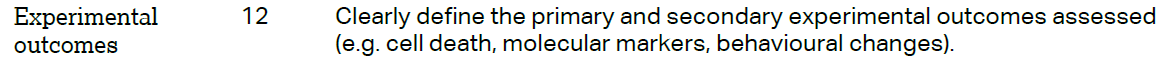 | Paragraph 7 |  |
| 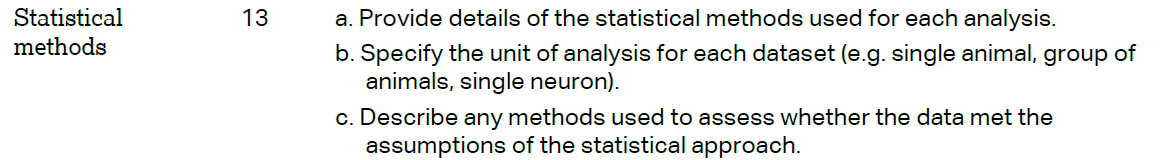 | Paragraph 8 |  |
| RESULTS |  |  |
| 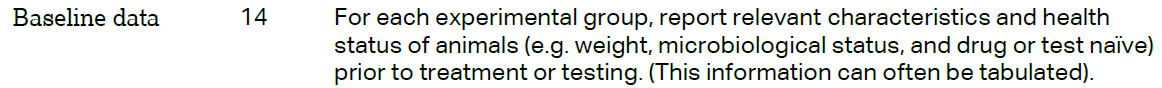 | Methods Paragraph 2 |  |
| 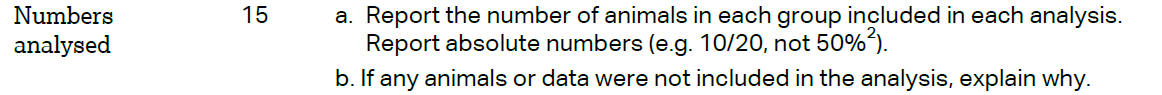 | Methods Paragraph 2 |  |
| 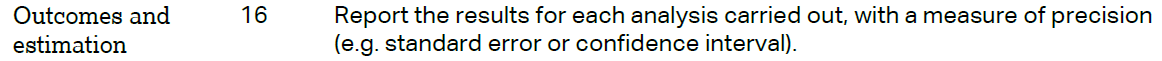 | Methods Paragraph 7, 8 |  |
| 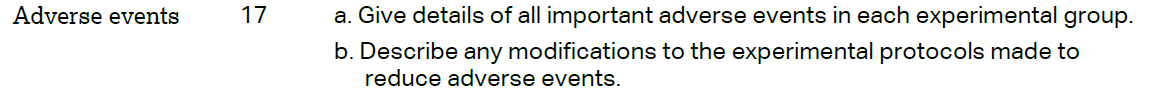 |  |  |
| DISCUSSION |  |  |
| 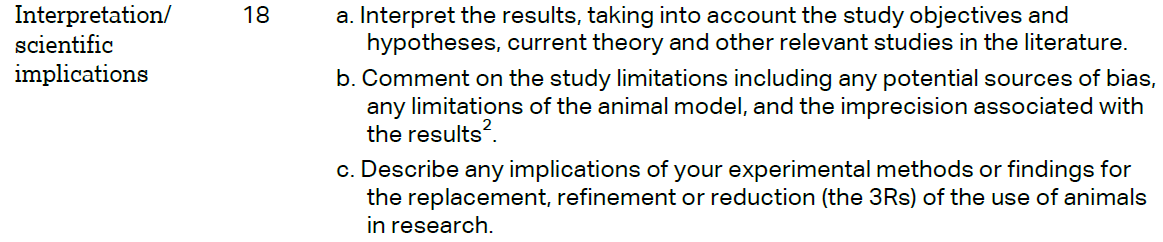 | Throughout  Paragraph 8  Paragraph 3 |  |
| 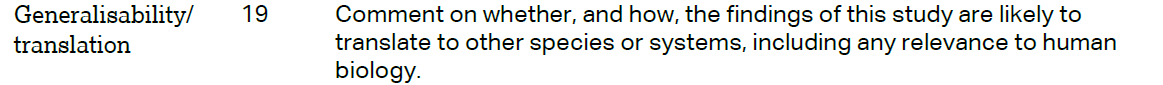 | Paragraph 10 |  |
| 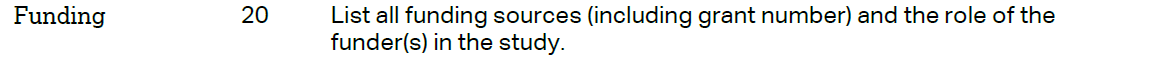 | | Title page |


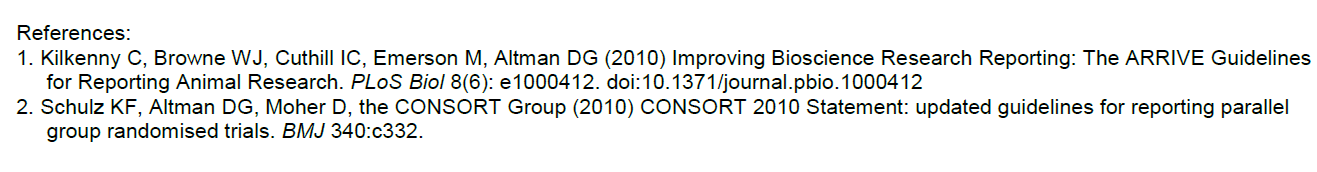

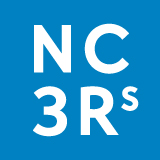

Supplement: S1 File — (DOCX) [file pone.0181457.s001.docx]
